# Evaluation in mice of *Brucella ovis* attenuated mutants for use as live vaccines against *B. ovis* infection

**DOI:** 10.1186/1297-9716-45-61

**Published:** 2014-06-04

**Authors:** Pilar Sancho, Carmen Tejedor, Rebeca S Sidhu-Muñoz, Luis Fernández-Lago, Nieves Vizcaíno

**Affiliations:** 1 Departamento de Microbiología y Genética, Edificio Departamental, Universidad de Salamanca, Plaza Doctores de la Reina s/n, 37007 Salamanca, Spain; 2 Instituto de Investigación Biomédica de Salamanca (IBSAL), Hospital Universitario de Salamanca, Pº de San Vicente 52-182, 37007 Salamanca, Spain

## Abstract

*Brucella ovis* causes ram contagious epididymitis, a disease for which a specific vaccine is lacking. Attenuated *Brucella melitensis* Rev 1, used as vaccine against ovine and caprine brucellosis caused by *B. melitensis*, is also considered the best vaccine available for the prophylaxis of *B. ovis* infection, but its use for this purpose has serious drawbacks. In this work, two previously characterized *B. ovis* attenuated mutants (Δ*omp25*d and Δ*omp22*) were evaluated in mice, in comparison with *B. melitensis* Rev 1, as vaccines against *B. ovis*. Similarities, but also significant differences, were found regarding the immune response induced by the three vaccines. Mice vaccinated with the *B. ovis* mutants developed anti-*B. ovis* antibodies in serum of the IgG_1_, IgG_2a_ and IgG_2b_ subclasses and their levels were higher than those observed in Rev 1-vaccinated mice. After an antigen stimulus with *B. ovis* cells, splenocytes obtained from all vaccinated mice secreted similar levels of TNF-α and IL12(p40) and remarkably high amounts of IFN-γ, a crucial cytokine in protective immunity against other *Brucella* species. By contrast, IL-1α -an enhancer of T cell responses to antigen- was present at higher levels in mice vaccinated with the *B. ovis* mutants, while IL-10, an anti-inflammatory cytokine, was significantly more abundant in Rev 1-vaccinated mice. Additionally, the *B. ovis* mutants showed appropriate persistence, limited splenomegaly and protective efficacy against *B. ovis* similar to that observed with *B. melitensis* Rev 1. These characteristics encourage their evaluation in the natural host as homologous vaccines for the specific prophylaxis of *B. ovis* infection.

## Introduction

Brucellosis caused by *Brucella ovis* mainly induces chronic epididymitis and orchitis in rams. The disease is widely distributed and constitutes one of the most important causes of reproductive failure in sheep [1]. In addition to a reduction in male fertility, *B. ovis* induces occasional abortions in ewes and increases the risk of perinatal mortality and low weight in lambs, thus having an important economic impact in the livestock sector [1].

*Brucella* strains are smooth (S) or rough (R), depending on the presence or absence of O-polysaccharide (O-PS) chains in the lipopolysaccharide (LPS) respectively. Other outer membrane (OM) components -such as OM proteins (OMPs) or the core and lipid A of LPS- are masked by O-PS in S strains, while in R brucellae, such as *B. ovis,* they are exposed on the bacterial surface [2]. In infected animals and humans, the O-PS chains induce a strong antibody response that constitutes the basis for the serological diagnosis of infections caused by S *Brucella* strains [2].

*Brucella melitensis* Rev 1 is an S live attenuated strain currently used for vaccination campaigns against ovine and caprine brucellosis, which is almost exclusively caused by *B. melitensis* and *B. ovis*. It is also considered the best vaccine available for the prophylaxis of *B. ovis* infection despite being mainly used for preventing *B. melitensis* infection [3-7]. However, several drawbacks are associated with Rev 1 vaccine, such as its variable efficacy, the induction of antibodies against S-LPS interfering with the diagnosis of brucellosis caused by *B. melitensis* and other S brucellae, the ability to induce human infections and abortions in animals and its resistance to streptomycin [5,8,9]. Additionally, due to its serological cross-reactivity with other S *Brucella* strains, it is banned in countries where *B. melitensis* has been eradicated, some of which are important sheep producers and where *B. ovis* infection constitute a serious problem. Therefore, the development of a new vaccine able to substitute *B. melitensis* Rev 1 strain in the prophylaxis of *B. ovis* infection is a matter of great interest.

Subcellular vaccines based on the hot saline extract (HS) of *B. ovis* -mainly composed of R-LPS and OMPs- have been extensively analyzed in mice and rams but their protective activity is limited [10-14]. Among these vaccines, HS encapsulated in microparticles has provided the best results in terms of protection [12]. R-LPS and/or recombinant proteins, either as purified antigens or as DNA vaccines requiring repeated inoculations, have also been assayed, with different success rates [13-17]. Considering that the best vaccines available against brucellosis caused by S *Brucella* strains are homologous S live attenuated strains [18], the development of an R *B. ovis* attenuated vaccine strain seems to be an interesting approach for the control of *B. ovis* infection.

In light of the above, the aim of this work was to evaluate the immunogenicity and protective activity against *B. ovis* infection of two *B. ovis* attenuated mutants obtained previously by inactivation of the genes coding for Omp25d and Omp22 [19], two OMPs of the Omp25/Omp31 family [20,21]. Considering that routine analysis of *Brucella* vaccines in the natural host is precluded due to economical and practical difficulties [22], the murine model was selected for this purpose since it is proposed by the World Organisation for Animal Health (OIE) as a method for the evaluation of *Brucella* vaccines administered in sheep [23] and it is currently used in brucellosis research for the assessment of the protective activity of vaccines [22,24]. Additionally, a good correlation between the mouse model and sheep has been observed for other *Brucella* vaccines [25] and the *B. ovis* attenuated mutants have been analyzed in parallel with *B. melitensis* Rev1 -the attenuated strain used in official vaccination campaigns against sheep brucellosis [3,23]- that constitutes a control of recognized vaccine efficacy in the natural host.

## Materials and methods

### Bacterial strains and culture conditions

The *Brucella* strains used in this work are shown in Table 1. The R virulent *B. ovis* PA strain and smooth (S) attenuated *B. melitensis* Rev 1 classical vaccine were obtained from the Institut National de la Recherche Agronomique, Nouzilly, France. Attenuated *B. ovis* PNV25dA (Δ*omp25d*) and *B. ovis* PNV22A (Δ*omp22*) are mutant strains derived from virulent *B. ovis* PA [19]. They were obtained previously by substitution of *omp25d* or *omp22*, respectively, by a kanamycin (Kan) resistance gene [19].

**Table 1:** ***Brucella *** **strains used in this work**

**Strain**	**Origin**^**a**^	**Virulence (relevant genotype)**	**LPS phenotype**	**Relevant antibiotic resistance**
*B. melitensis* Rev 1	BCCN V4a	Classical attenuated vaccine	Smooth	Streptomycin
*B. ovis* PA	BCCN 76-250	Virulent strain	Rough	-
*B. ovis* PNV25dA	[19]	Recombinant *B. ovis* PA attenuated mutant (Δ*omp25d*)	Rough	Kanamycin
*B. ovis* PNV22A	[19]	Recombinant *B. ovis* PA attenuated mutant (Δ*omp22*)	Rough	Kanamycin

^a^BCCN, *Brucella* Culture Collection, Nouzilly, France.

All *Brucella* strains were propagated in tryptic soy agar (Pronadisa-Laboratorios Conda, Torrejón de Ardoz, Spain) supplemented with 0.3% yeast extract (Pronadisa-Laboratorios Conda) and 5% horse serum (Gibco-Life Technologies, Grand Island, USA) (TSA-YE-HS). When appropriate, depending on the resistance profile of each *Brucella* strain (Table 1), streptomycin (Strep) or Kan (Sigma-Aldrich, St. Louis, USA) was added to TSA-YE-HS medium to a final concentration of 50 μg/mL. Bacteria were incubated at 37 °C in a 5% CO_2_ atmosphere.

### Mice and inoculation procedure

Female 6-week-old BALB/c mice (Charles River Laboratories, Chatillon-sur-Chalaronne, France), received at our laboratory one week previously, were used. They were randomly distributed into experimental groups and kept with water and food *ad libitum* in the animal experimentation facilities of the University of Salamanca (registration number PAE SA-001). Procedures with mice were designed according to Spanish and European legislation regarding the use of animals in research (RD 1201/05 and directive 2010/63/UE).

Vaccination and challenge with *Brucella* strains were performed by intraperitoneal inoculation of bacterial suspensions in phosphate-buffered saline (PBS; pH 7.2) prepared from fresh cultures incubated for 44 h. The number of colony-forming units per mL (CFU/mL) was estimated by optical density (OD) readings at 600 nm (OD_600_) and the exact doses administered were determined retrospectively by triplicate plating of the properly diluted inocula on TSA-YE-HS.

### Immunization of mice and sample collection for evaluation of the immune response and vaccine strain persistence

Mice were inoculated with PBS (unvaccinated control), with the *B. melitensis* Rev 1 classical vaccine (10^5^ CFU), or with the Δ*omp25d* (10^7^ CFU) or Δ*omp22* (10^8^ CFU) mutants of *B. ovis* PA. The dose of vaccination for *B. melitensis* Rev 1 was that commonly used for vaccine studies in mice [22], while the vaccination doses for the *B. ovis* attenuated mutants were selected according to previous results of spleen colonization when they were inoculated at a dose of 5 × 10^6^ CFU/mice [19] and taking into account vaccine studies with other *Brucella* species [22]. Thus, considering that the Δ*omp22* mutant was severely attenuated in that previous study [19], the dose of 10^8^ CFU/mice was selected to analyze its vaccine properties, while vaccination with the Δ*omp25d* mutant -that showed a reduced persistence but a high level of spleen colonization at week 1 post-inoculation (pi) (about 2 log units higher than the parental strain) [19]- was performed at a dose of 10^7^ CFU/mice.

Sera for analysis of the antibody response were obtained from submandibular blood extracted from five mice per group at selected time-points. The same mice were then euthanized and their spleens isolated in order to determine both the CFU number of the vaccine strain (persistence assay) and the cytokine profile.

To evaluate the ability of splenocytes from vaccinated mice to secrete cytokines in response to a stimulus with heat-inactivated *B. ovis* PA whole cells, five mice per group were immunized as described above and their spleens were removed 28 days later for further ex vivo analysis of spleen cell cultures, as detailed below.

### Evaluation of vaccine strain persistence

Persistence of the vaccine strains in mice was evaluated by determining the bacterial CFU in spleen at 1, 3, 7, 14, 21, 28 and 49 days post-inoculation (dpi). At each selected pi time-point, spleens from five mice per group were weighed and individually homogenized in 9 vol of Hanks’ balanced solution (Gibco-Life Technologies). Ten-fold serial dilutions were plated onto TSA-YE-HS to determine bacterial counts. The results were expressed as means ± SD (*n* = 5) of the log CFU/spleen for each strain. For each vaccination group, the mean ± SD (*n* = 5) of the spleen weight in grams was also calculated.

### Evaluation of the antibody response in serum against whole cells of *B. ovis*

Serum immunoglobulin (Ig) G (IgG) titers in immunized mice were determined by indirect enzyme-linked immunosorbent assay (i-ELISA) at 1, 3, 7, 14, 21, and 28 dpi (pre-challenge period in protection experiments). Plates (96-well Maxisorp, Nunc-Thermo Fisher Scientific, Roskilde, Denmark) were coated by overnight incubation with 100 μL of *B. ovis* PA suspensions (OD_600_ = 1) prepared in PBS from heat-inactivated (1 h, 65 °C) fresh cultures (44 h of growth). The wells were then incubated sequentially with 5% skim milk in PBS (30 min at 37 °C), double-serial dilutions of mouse sera in PBS containing 0.05% Tween 20 (PBS-T; 1 h at 37 °C) and goat anti-mouse IgG (Fc)-peroxidase conjugate (Sigma-Aldrich) diluted 1:4000 in PBS-T (1 h at room temperature). The different steps were separated by three washes with 0.9% NaCl, 0.05% Tween 20. Finally, the substrate solution –1 mM 2,2’-azino-di-(3-3-ethylbenzothiazoline-sulfonic acid) (ABTS; Sigma-Aldrich) and 2 mM H_2_O_2_ (Sigma-Aldrich) in 0.1 M citrate, pH 4.2- was added to the wells and the OD_405_ readings were recorded on a Microplate Reader 550 (Bio-Rad, Hercules, USA) after 30 min incubation at room temperature. IgG isotypes were determined under the same conditions but using goat anti-mouse IgG_1_-, IgG_2a_- or IgG_2b_-peroxidase conjugates (Santa Cruz Biotechnology, Dallas, USA).

Antibody titers in serum were defined as the inverse of the highest serum dilution scoring an OD_405_ value two times higher than that obtained with the blank (mean OD_405_ of six wells in which serum was replaced by dilution buffer). The results for IgG were represented as means ± SD of the log of the titers obtained with five mice analyzed individually. The results concerning IgG isotypes were represented as means ± SD of the OD_405_ values obtained with a 1/100 dilution of a pool of the five sera assayed in triplicate.

### Evaluation of cytokines in spleen and in splenocyte cultures

Cytokine levels in the spleens of immunized mice were evaluated at 1, 3, 7, 14, 21 and 28 dpi (pre-challenge period in protection experiments). CHAPS (Sigma-Aldrich) was added (1% final concentration) to mouse spleens homogenized in Hanks balanced salt solution (Gibco-Life Technologies) (see vaccine persistence section) [26,27]. After a 1-h incubation at 4 °C, cell debris was removed by centrifugation and the supernatants were stored at −80 °C until analysis. The levels of interferon-γ (IFN-γ), tumor necrosis factor-α (TNF-α), interleukin-1α (IL-1α), IL-10 and IL-12(p40) were quantified by sandwich ELISA with OptEIA^TM^ Mouse Sets specific to each cytokine, as instructed by the manufacturer (BD Biosciences, San Diego, USA) and described elsewhere [28]. The results for each vaccination group were expressed as means ± SD of the cytokine quantity (ng) detected in the spleens of five individual mice at each time-point.

Cytokine production was also evaluated, after stimulation with heat-inactivated *B. ovis* PA whole cells, in cell cultures of splenocytes obtained from immunized mice at 28 dpi. Uniform single-cell suspensions were prepared by gentle disruption of spleens, as described previously [28]. Cells were suspended in complete RPMI medium (RPMI supplemented with 10% fetal calf serum, 4 mM L-glutamine, 1 mM sodium pyruvate, 0.05 mM 2-mercaptoethanol (Gibco-Life Technologies) and 100 μg/mL gentamicin), distributed in 24-well plates (4 × 10^5^ cells/well in 1 mL) and cultured at 37 °C and a 5% CO_2_ atmosphere. Splenocytes were stimulated by exposure to heat-inactivated (1 h at 65 °C) *B. ovis* PA whole cells (10^7^ CFU/well), 10 μg/mL of the mitogen concanavalin A (Sigma-Aldrich) as a positive control of cell proliferation, or culture medium as a negative control. The stimulating agents were prepared in complete RPMI medium and added to each well in 100-μL volumes. After 72 h, the supernatants were harvested, centrifuged and stored at –80 °C until use for cytokine quantification, performed as described above. Two wells were used for each experimental condition and mouse. The results for each vaccination group were expressed as means ± SD of the cytokine amount (ng/well) in the supernatants of splenocytes obtained from five individual mice. The results obtained with the positive and negative controls (concanavalin A and RPMI as stimulating agents, respectively) were as expected and are not shown.

### Protection studies in mice

Mice inoculated with PBS (unvaccinated control), *B. melitensis* Rev 1 (10^5^ CFU/mouse), *B. ovis* Δ*omp25d* (10^7^ CFU/mouse) or *B. ovis* Δ*omp22* (10^8^ CFU/mouse) were challenged 28 days later with 10^5^ CFU of virulent *B. ovis* PA. Three weeks after the challenge (49 days after immunization), the bacterial CFU numbers in spleen were counted in five mice from each experimental group, as described previously [19]. The CFU number of virulent *B. ovis* PA in mice vaccinated with *B. melitensis* Rev 1 (Strep-resistant strain; Table 1) was obtained by subtracting the CFU obtained in TSA-YE-HS-Strep medium from those obtained in the same medium without antibiotic. Similarly, differential counts in medium with and without Kan were used to determine the CFU of the challenge strain in the spleens of mice vaccinated with the *B. ovis* attenuated strains. Results were expressed as means ± SD (*n* = 5) of the log CFU/spleen of *B. ovis* PA for each vaccination group.

### Statistical analyses

Statistical comparisons between means were performed using analysis of variance. The levels of significance of the differences between the experimental groups were determined with the post-hoc Fisher’s protected least significant differences (PLSD) test.

## Results

### Persistence of vaccine strains in spleen

Spleen weight and bacterial counts were determined at several pi time-points in mice inoculated with *B. melitensis* Rev 1, *B. ovis* Δ*omp25d*, and *B. ovis* Δ*omp22* (retrospectively determined doses of 0.9 × 10^5^, 1.2 × 10^7^, and 0.80 × 10^8^ CFU/mouse, respectively).

**Figure 1:**
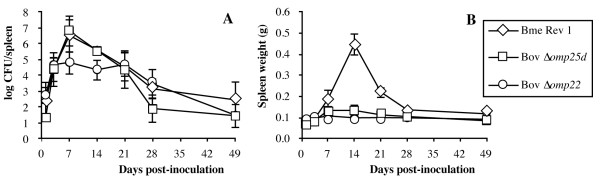
**Kinetics of splenic infection and spleen weight in mice inoculated with the vaccines. ***B. melitensis* Rev 1 (Bme Rev 1; 1 × 10^5^ CFU/mouse), *B. ovis* PNV25dA (Bov Δ*omp25d*; 1 × 10^7^ CFU/mouse), and *B. ovis* PNV22A (Bov Δ*omp22*; 1 × 10^8^ CFU/mouse) were used as vaccines. Results are expressed as means ± SD (*n* = 5) of the log CFU/spleen **(A)** or spleen weight **(B)** at each time-point.

Under the assay conditions, spleen colonization by *B. melitensis* Rev1 and *B. ovis* Δ*omp25d* was quite similar, with a peak of about 7 log CFU/spleen at 1 week pi and a progressive decrease in bacterial counts thereafter until the end of the experiment on day 49 pi (Figure 1A). However, while the classic *B. melitensis* Rev 1 vaccine was detected in most of the animals on days 28 and 49 pi, all mice inoculated with the *B. ovis* Δ*omp25d* mutant had cleared the infection by day 49 pi (according to the detection limit of the technique) and only 1 out 5 five mice showed bacteria in its spleen at day 28 pi. With the *B. ovis* Δ*omp22* attenuated mutant, the highest level of spleen colonization extended from day 3 to day 21 pi and was 2 log units lower (about 5 log CFU/spleen) than that obtained with the two other vaccines on day 7 pi. Thereafter, *B. ovis* Δ*omp22* behaved similarly to the other vaccines (Figure 1A).

Regarding spleen weight (Figure 1B), the two *B. ovis* attenuated mutants did not induce significant inflammation of the spleen along the assay, while the *B. melitensis* Rev 1 classic vaccine induced a strong transient splenomegaly, reaching its highest level at 14 dpi. At that moment, spleen weight in the mice inoculated with *B. melitensis* Rev 1 was about 4 times higher than that observed in mice inoculated with the *B. ovis* mutants (Figure 1B).

### Antibody response against *B. ovis* induced by the vaccines

The levels of serum IgG able to react with whole *B. ovis* cells in i-ELISA were evaluated up to day 28 pi (pre-challenge period in the protection experiments) in mice inoculated, as described for the persistence assay, with PBS (negative control group) or each of the three attenuated vaccines.

**Figure 2:**
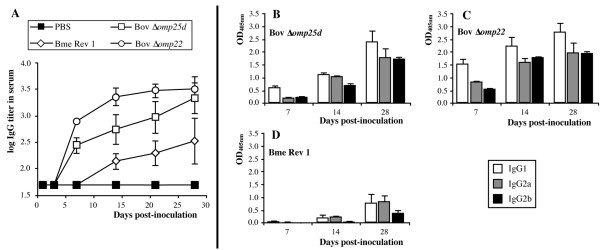
**Antibodies, reactive with whole *B. ovis *PA cells, in serum.** Mice were inoculated with PBS or the *B. melitensis* Rev 1, *B. ovis* PNV25dA, and *B. ovis* PNV22A vaccine strains. IgG levels are expressed as means ± SD (*n* = 5) of the log of IgG titers in i-ELISA at each time-point **(A)**. IgG_1_, IgG_2a_ and IgG_2b_ levels were represented as the OD_405_ values obtained in i-ELISA with a 1/100 dilution of a pool of five sera (three repeats) from mice inoculated with *B. ovis* PNV25dA **(B)**, *B. ovis* PNV22A **(C)** or *B. melitensis* Rev 1 **(D)**. See strain abbreviations and inoculation doses in legend to Figure 1.

In mice immunized with the two *B. ovis* attenuated mutants, the IgG response against whole *B. ovis* cells became evident by day 7 pi and increased progressively thereafter until the end of the experiment (Figure 2A). On days 7 and 14 pi, IgG titers were higher in mice immunized with *B. ovis* Δ*omp22* than in those immunized with *B. ovis* Δ*omp25d*, but they reached similar levels by day 28 pi (Figure 2A). In comparison, the IgG response in mice immunized with *B. melitensis* Rev 1 was delayed and weaker, since detectable titers appeared on day 14 pi and were lower than those observed with the *B. ovis* vaccines at all time-points checked (Figure 2A). Inside each vaccination group, no important differences were detected in the proportion of the IgG_1_, IgG_2a_ and IgG_2b_ isotypes (Figure 2B-D).

### Spleen cytokines in vaccinated mice

The cytokine profile in spleen was also evaluated until day 28 pi in mice inoculated, as described for the persistence assay, either with PBS or with the attenuated strains.

**Figure 3:**
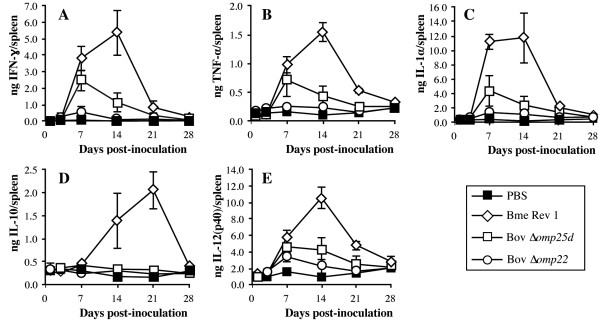
**Levels of cytokines in spleen.** IFN-γ **(A)**, TNF-α **(B)**, IL-1α **(C)**, IL-10 **(D)**, and IL-12(p40) **(E)** were evaluated in the spleens of mice inoculated with PBS or the *B. melitensis* Rev 1, *B. ovis* PNV25dA, and *B. ovis* PNV22A vaccine strains. See strain abbreviations and inoculation doses in legend to Figure 1. Results are expressed as means ± SD (*n* = 5) of each cytokine amount in spleen at each time-point.

The highest levels of IFN-γ, TNF-α, IL-1α, IL-10 and IL-12(p40) in spleen were detected in mice immunized with *B. melitensis* Rev 1 (Figure 3). In this group of mice, all cytokines, except IL-10, were present in high levels on days 7 and 14 pi, with a production peak on day 14 pi, and a decrease thereafter until day 28 pi, when all cytokines had levels similar to those detected in mice inoculated with PBS (Figure 3). IL-10 production in the Rev 1-vaccinated mice followed a similar pattern but with a delay of 7 days, the highest levels being detected on days 14 and 21 pi (Figure 3D). Whereas TNF-α and IL-10 scored the lowest levels (about 1.5 and 2 ng/spleen, respectively), the strongest response was obtained with IL-1α and IL-12(p40) (maximum levels about 10 ng/spleen) followed by IFN-γ (5 ng/spleen).

Although to a lesser extent than *B. melitensis* Rev 1, *B. ovis* Δ*omp25d* also induced the production in spleen of all cytokines tested (Figure 3), with the exception of IL-10 (Figure 3D). A peak of cytokine production was observed on day 7 pi, which was followed by a progressive decrease until day 28 pi, when no differences between the mice inoculated with *B. ovis* Δ*omp25d* and the PBS-inoculated mice were observed (Figure 3).

In general, the mice immunized with *B. ovis* Δ*omp22* did not show statistically significant differences in cytokine production in spleen when compared with mice inoculated with PBS (Figure 3). The exception was IL-12(p40) on day 7 pi, whose concentration in spleen was slightly higher in mice inoculated with *B. ovis* Δ*omp22* (Figure 3E).

### Cytokine secretion by splenocytes of vaccinated animals in response to a stimulus with *B. ovis* PA whole cells

Mice were immunized with *B. melitensis* Rev1, *B. ovis* Δ*omp25d, B. ovis* Δ*omp22* (retrospectively determined doses of 0.9 × 10^5^, 1.1 × 10^7^, and 1.4 × 10^8^ CFU/mouse, respectively) or with PBS.

To evaluate the ability of spleen cells of the vaccinated animals to respond to a second stimulus by antigen, splenocytes obtained on day 28 pi were stimulated with heat-inactivated *B. ovis* PA and the cytokines secreted were quantified after 72 h of incubation (see Materials and Methods).

**Figure 4:**
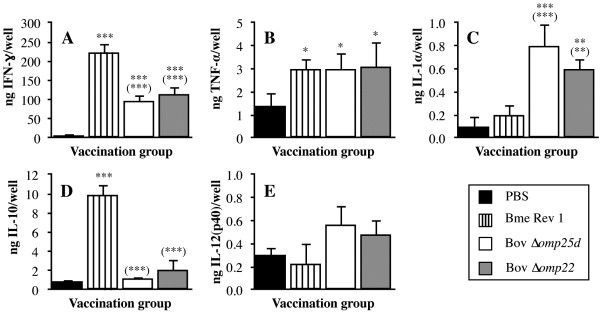
**Cytokine production by stimulated splenocytes obtained from mice inoculated with PBS or the vaccine strains.** Mice were inoculated with PBS or with *B. melitensis* Rev 1, *B. ovis* PNV25dA, and *B. ovis* PNV22A vaccine strains. Spleen cells were obtained 28 days after vaccine inoculation from five mice per group. Splenocytes were cultured as described in Materials and methods and stimulated with heat-killed *B. ovis* PA (10^7^ CFU/well) for 72 h. Supernatants were analyzed by ELISA tests specific for IFN-γ **(A)**, TNF-α **(B)**, IL-1α **(C)**, IL-10 **(D)**, and IL-12(p40) **(E)**. Results are expressed as means ± SD (*n* = 5) of each cytokine amount per well. See strain abbreviations and inoculation doses in legend to Figure 1. Significant differences (PLSD test) between mice inoculated with each vaccine and control mice inoculated with PBS are marked as follows: ***, *P* ≤ 0.0005; **, *P* ≤ 0.005; *, *P* ≤ 0.05. Significant differences between mice inoculated with the *B. ovis* attenuated strains and mice vaccinated with *B. melitensis* Rev 1 are represented in brackets following the same rule.

Splenocytes from mice immunized with *B. melitensis* Rev 1 or with the two *B. ovis* attenuated mutants secreted high levels of IFN-γ (ranging between 97 and 223 ng/well), which was the most abundant cytokine, in response to the second antigen stimulus with *B. ovis* PA (Figure 4A). By contrast, IFN-γ production by splenocytes obtained from mice inoculated with PBS was comparatively weak (about 5 ng/well; *P* < 0.0005) (Figure 4A).

The three attenuated vaccines induced the secretion of TNF-α by stimulated spleen cells to a similar extent and in amounts that were two times higher (*P <* 0.05) than those detected in the group inoculated with PBS (Figure 4B). Regarding the production of IL-1α, no differences were found between the stimulated splenocytes of mice immunized with *B. melitensis* Rev 1 and those of the control mice inoculated with PBS (Figure 4C). In contrast, the production of IL-1α in the *B. ovis* Δ*omp25d* and *B. ovis* Δ*omp22* vaccination groups was about four times and three times higher, respectively (Figure 4C).

A strong IL-10 response was detected in stimulated spleen cells of mice immunized with the *B. melitensis* Rev 1 classic vaccine (Figure 4D), while the spleen cells of mice vaccinated with the two *B. ovis* attenuated strains behaved similarly to those obtained from mice immunized with PBS (Figure 4D). Regarding the secretion of IL-12(p40) by stimulated spleen cells, no statistically significant differences were found between the mice immunized with the attenuated *Brucella* strains and the control mice immunized with PBS (Figure 4E).

### Protective efficacy of the attenuated mutants against *B. ovis* infection

Mice immunized with PBS (negative immunization control), *B. melitensis* Rev 1, *B. ovis* Δ*omp25d* or *B. ovis* Δ*omp22* (retrospectively determined doses of 0.9 × 10^5^, 1.1 × 10^7^, and 1.4 × 10^8^ CFU/mouse, respectively) were challenged 28 days after immunization with virulent *B. ovis* PA (retrospectively determined dose of 0.9 × 10^5^ CFU/mouse). Bacteria were counted in spleen 3 weeks after the experimental infection.

**Figure 5:**
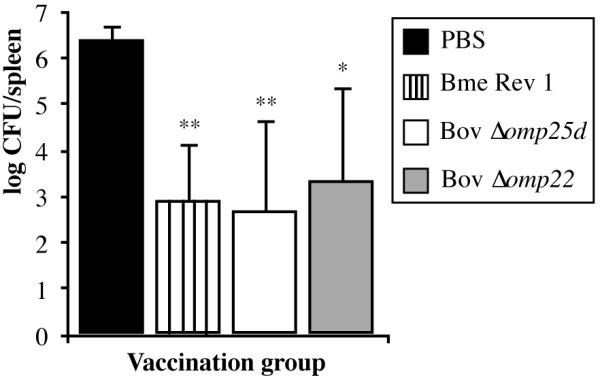
**Protection conferred in mice against virulent *B. ovis *PA by the *Brucella *vaccines.** Mice were inoculated with PBS (negative control), *B. melitensis* Rev 1, *B. ovis* PNV25dA, or *B. ovis* PNV22A (see strain abbreviations and inoculation doses in legend to Figure 1) and were challenged 28 days later with 10^5^ CFU/mouse of virulent *B. ovis* PA. Three weeks after infection, the CFUs of *B. ovis* PA in spleen were counted in five mice per vaccination group. Results are expressed as means ± SD (*n* = 5) of the log CFU/spleen of *B. ovis* PA. Significant differences (PLSD test) between vaccination groups are marked as described in the legend to Figure 4.

The *B. melitensis* Rev 1 classic vaccine and the two *B. ovis* attenuated strains conferred similar protection (*P* > 0.05) against a challenge with *B. ovis*. When compared to mice inoculated with PBS, the three attenuated vaccines reduced spleen colonization of virulent *B. ovis* PA in the order of 3 log units (Figure 5). Only the challenge strain was isolated in mice vaccinated with *B. ovis* Δ*omp25d* while the *B. ovis* Δ*omp22* and *B. melitensis* Rev 1 vaccine strains were detected in 3 out 5 mice.

## Discussion

*B. melitensis* Rev 1 is considered the best vaccine available against *B. ovis* infection but it has important drawbacks that constrain its use for the specific prophylaxis of brucellosis caused by *B. ovis*[3-7]. Since live attenuated vaccines have provided the best results in terms of protection against infections caused by other *Brucella* species [29-31], it is to be expected that an attenuated strain would also be the best choice for a vaccine targeting *B. ovis* infection. Smooth vaccine strains other than *B. melitensis* Rev 1 (i.e. *B. abortus* S19 or *B. suis* S2) would not avoid the interference with the serological diagnosis of smooth strain infections and, consequently, are not suitable vaccines. Theoretically, rough *B. abortus* RB51 would offer a good alternative because interference with diagnosis would be minimized. However, in spite of the rough surface phenotype of *B. abortus* RB51, the risk of inducing O-PS antibodies cannot be ignored owing to the presence of small amounts of O-PS in its cytoplasm [32] and, more importantly, this attenuated vaccine proved to be inefficient in preventing against *B. ovis* infection in rams [6]. Moreover, although some cases of heterologous protection have been reported, in livestock homologous *Brucella* spp. are usually more protective than heterologous *Brucella* spp. [31]. Accordingly, a *B. ovis* attenuated strain would likely be the most interesting candidate to develop a specific vaccine against ram contagious epididymitis. This hypothesis also seems to be supported by the fact that *B. ovis* displays significant differences in surface topology, outer membrane properties and antigenic behavior when compared to other *Brucella* species [2,21,33-35].

Although a recent work has reported the ability of a *B. ovis* attenuated mutant to induce an immune response in a natural host [36] and another work has analyzed the protective activity of a *B. ovis* mutant in a murine model [37], the potential of attenuated *B. ovis* strains as vaccines remains almost unexplored. Two *B. ovis* mutants defective in Omp25d and Omp22 surface protein, have previously been shown to display attenuated virulence in a mouse model [19,38] and in the present work they were evaluated as homologous live attenuated vaccines, in comparison to heterologous *B. melitensis* Rev 1, for the prophylaxis of *B. ovis* infection.

In a previous study, when inoculated at a dose of 5 × 10^6^ CFU/mouse the Δ*omp22* mutant of *B. ovis* PA was almost undetectable in spleen from week 1 pi [19], while the Δ*omp25d* mutant displayed a more moderate attenuation, with even higher spleen colonization during the initial stages of infection than that of the parental strain but with a rapid clearance thereafter [19]. However, when the immunization dose was increased to 1 × 10^8^ CFU/mouse, even though maximum bacterial counts were 2 log units lower than those of *B. melitensis* Rev 1, the persistence in spleen of *B. ovis* Δ*omp22* was similar to that observed for the Rev 1 vaccine (Figure 1A) at the dose commonly used for protection studies in mouse models [22] and also in this work. The increase in the inoculation dose of the Δ*omp25d* mutant led to a spleen growth curve that exhibited the same pattern as that observed for the classic vaccine *B. melitensis* Rev 1 under our assay conditions of (Figure 1A). Accordingly, the requirement of a certain degree of persistence considered necessary for an efficacious vaccine against *Brucella* spp. [39,40] seems to have been accomplished with the *B. ovis* Δ*omp25d* and Δ*omp22* mutants, since their evolution in spleen was similar to that observed with a recognized control of vaccine efficacy against *B. ovis* (Figure 1A).

*B. melitensis* Rev 1 induced a strong inflammatory response in spleen, this response reaching its highest level of intensity on day 14 pi, while *B. ovis* Δ*omp25d*, with a spleen colonization profile similar to that of *B. melitensis* Rev 1 (Figure 1A), and *B. ovis* Δ*omp22* only elicited a moderate degree of splenomegaly (Figure 1B). Spleen inflammation in the mice inoculated with *B. melitensis* Rev 1 correlated with the high levels of cytokines detected in this organ, since with the exception of IL-10 all the cytokines peaked on day 14 pi (Figure 3), concomitantly with spleen weight (Figure 1B). In these mice, the maximum spleen levels of IL-10 were reached one week later (Figure 3D), which, considering the anti-inflammatory role proposed for this cytokine, could reflect a mechanism aimed at counterbalancing the deleterious effects in the host derived from the exacerbated inflammatory response [41,42]. This hypothesis seems to be supported by the fact that the increase in IL-10 observed in mice vaccinated with *B. melitensis* Rev 1 (Figure 3D) was accompanied by a striking reduction in both spleen weight (Figure 1B) and the amount of the other cytokines analyzed (Figure 3). The reduction in cytokine levels continued until day 28 post-vaccination, when they reached the basal values observed in mice inoculated with PBS or with the *B. ovis*-derived vaccines (Figure 3). Since the challenge with virulent *B. ovis* PA was performed at that moment, the protection afforded by the vaccines cannot be attributed to residual cytokines from the primary immunization. However, vaccination with *B. melitensis* Rev 1 or the attenuated Δ*omp22* and Δ*omp25d* strains of *B. ovis* induced a memory immune response that could be activated by *B. ovis* cells and that was evidenced by the pattern of cytokine secretion when splenocytes obtained from vaccinated mice were re-stimulated ex vivo with killed *B. ovis* PA whole cells (Figure 4).

The strong inflammatory response -revealed by the prominent increase in spleen weight and cytokine levels- detected in the spleens of mice vaccinated with *B. melitensis* Rev 1 with respect to that induced by the *B. ovis* vaccines (Figures 1B and 2) would initially predict a better protective activity for the former vaccine. However, the protection conferred by *B. ovis* Δ*omp25d* and Δ*omp22* against an experimental *B. ovis* infection was equivalent to that of *B. melitensis* Rev 1 (Figure 5), and several reasons can be invoked to explain this. First, the higher response of antibodies able to bind to *B. ovis* whole cells detected in mice immunized with the *B. ovis*-derived vaccines (Figure 2) constitutes an advantageous characteristic of the homologous vaccines over *B. melitensis* Rev 1, since a more prominent role for antibodies than for T lymphocytes has been identified in protective immunity against *B. ovis*[43]. Second, the level of IFN-γ, which is known to play a critical role in the control of primary and secondary *Brucella* infections [44,45], secreted by spleen cells from immunized mice in response to exposure to *B. ovis* whole cells was remarkably high (ranging between 97 and 223 ng/well) in the three vaccination groups (Figure 4A). Third, splenocytes from mice vaccinated with the *B. ovis*-derived vaccines secreted more IL-1α, which has been shown to be a potent enhancer of CD4 and CD8 T cell responses to antigen [46], than splenocytes obtained from mice vaccinated with *B. melitensis* Rev 1 or non-vaccinated mice (Figure 4C). Finally, re-stimulated spleen cells of mice vaccinated with the *B. ovis* attenuated mutants did not differ from those obtained from non-vaccinated mice regarding the production of IL-10, while vaccination with *B. melitensis* Rev 1 induced an intense IL-10 response in re-stimulated splenocytes. Considering the anti-inflammatory role proposed for this cytokine [41,42], this might also constitute an advantage for the *B. ovis* attenuated vaccines.

Some of these valuable characteristics of *B. ovis* Δ*omp25d* and Δ*omp22* are probably related to a more specific immune response induced when homologous vaccines are used. Despite the antigenic differences described for *B. ovis* in comparison with other *Brucella* species, only a moderate degree of antigenic diversity is expected between *B. melitensis* Rev 1 and *B. ovis* PA, considering the high degree of DNA identity shared by them [47]. However, S-LPS is the major immunodominant antigen in smooth strains such as *B. melitensis* Rev 1 [48,49], while R-LPS and OMPs are immunodominant components in rough *B. ovis* that lack S-LPS [50]. Accordingly, an important percentage of the immune response developed after vaccination with *B. melitensis* Rev 1 would probably not be effective against later infection by *B. ovis*. By contrast, the immune response triggered by the *B. ovis* attenuated vaccines would target homologous antigens during a *B. ovis* infection and would theoretically be more efficient in controlling the disease. Another positive aspect of *B. ovis* Δ*omp25d* and Δ*omp22* is that their protective activity is accompanied by a limited degree of splenomegaly, suggesting that these vaccines would prevent undesirable effects associated with inflammation in the host, as described for the classic vaccines *B. abortus* S19 and *B. melitensis* Rev 1 [51,52].

According to the results reported here, the attenuated strains *B. ovis* Δ*omp25d* and Δ*omp22* induce a solid immune response, with mixed Th1 and Th2 components, able to control later infection by *B. ovis* as efficiently as vaccination with heterologous *B. melitensis* Rev 1. Considering the drawbacks of vaccination with *B. melitensis* Rev 1 [5,8,9], especially for the prophylaxis of *B. ovis* infection, the attenuated vaccines analyzed here constitute a promising alternative as specific vaccines against ram contagious epididymitis that merits further evaluation in the natural host and the analysis of alternative formulations that might increase protective activity, such as microencapsulation for sustained release, as described for other *Brucella* vaccines [51,52].

## Competing interests

The authors declare that they have no competing interests.

## Authors’ contributions

PS, CT, LFL and NV conceived the study and participated in its design and coordination. PS, RSSM, and NV performed the experiments. PS, CT, LFL and NV wrote the manuscript. All authors read and approved the final manuscript.
